# Impaired Motor Skill Acquisition Using Mirror Visual Feedback Improved by Transcranial Direct Current Stimulation (tDCS) in Patients With Parkinson’s Disease

**DOI:** 10.3389/fnins.2019.00602

**Published:** 2019-06-19

**Authors:** Mitsuya Horiba, Yoshino Ueki, Ippei Nojima, Yoko Shimizu, Kento Sahashi, Shogo Itamoto, Ayuko Suzuki, Gohei Yamada, Noriyuki Matsukawa, Ikuo Wada

**Affiliations:** ^1^Department of Rehabilitation Medicine, Nagoya City University Graduate School of Medical Sciences, Nagoya, Japan; ^2^Department of Physical Therapy, Shinshu University Graduate School of Medicine, Nagano, Japan; ^3^Department of Neurology and Neuroscience, Nagoya City University Graduate School of Medical Sciences, Nagoya, Japan

**Keywords:** neuroplasticity, mirror visual feedback, rehabilitation, Parkinson’s disease, transcranial direct current stimulation

## Abstract

Recent non-invasive brain stimulation techniques in combination with motor training can enhance neuroplasticity and learning. It is reasonable to assume that such neuroplasticity-based interventions constitute a useful rehabilitative tool for patients with Parkinson’s Disease (PD). Regarding motor skill training, many kinds of tasks that do not involve real motor movements have been applied to PD patients. The purpose of this study is to elucidate whether motor skill training using mirror visual feedback (MVF) is useful to patients with PD in order to improve untrained hand performance dependent on the time course of training; and whether MVF combined with anodal transcranial direct current stimulation (tDCS) over primary motor cortex (M1) causes an additional effect based on increased motor cortical excitability. Eighteen right-handed patients with PD in the off-medication state and 10 age-matched healthy subjects (HS) performed four sessions of right-hand ball rotation using MVF (intervention) on two separate days, 1 week apart (day 1 and day 2). HS subjects received only sham stimulation. The intervention included four sessions of motor-skill training using MVF for 20 min comprised of four sets of training for 30 s each. PD patients were randomly divided into two intervention groups without or with anodal tDCS over the right M1 contralateral to the untrained hand. As the behavior evaluation, the number of ball rotations of the left hand was counted before (pre) and immediately after (post) intervention on both days (pre day 1, post day 1, pre day 2, and post day 2). Motor evoked potential (MEP), input-output function, and cortical silent period were recorded to evaluate the motor cortical excitatory and inhibitory system in M1 pre day 1 and post day 2. The number of ball rotations of the left hand and the facilitation of MEP by intervention were significantly impaired in patients with PD compared to HS. In contrast, if anodal tDCS was applied to right M1 of patients with PD, the number of ball rotations in accordance with I-O function at 150% intensity was significantly increased after day 1 and retained until day 2. This finding may help provide a new strategy for neurorehabilitation improving task-specific motor memory without real motor movements in PD.

## Introduction

Parkinson’s disease (PD) is a neurodegenerative disorder and some of its pathophysiology are related to the disruption in the dopaminergic system and the altered basal ganglia (BG)-thalamocortical circuitry (DeLong, 1990). Many kinds of rehabilitation which do not involve real motor movements have been applied to PD patients (Abbruzzese et al., 2015). In a motor imagery task, motor cortical excitability, and brain activation areas during hand action imagination were reduced and altered in PD patients (Thobois et al., 2000; Tremblay et al., 2008; Castiello et al., 2009). However, action observation training of finger movements improved the spontaneous rate of finger movements in PD patients (Pelosin et al., 2013). Moreover, PD patients showed action observation related facilitation of grasping movement only when the model was a Parkinsonian subject (Castiello et al., 2009). Based on these findings, fine visual input seems to be important for the improvement of motor dysfunction in PD patients without real motor training.

Mirror visual feedback (MVF) was first reported by Ramachandran et al. (1995) to alleviate phantom limb pain in amputees (Ramachandran et al., 1995). Since then, MVF has been successfully applied in patients with motor deficits especially due to stroke; motor training of the unimpaired limb with its MVF superimposed over the paretic limb led to a remarkable recovery (Altschuler et al., 1999; Yavuzer et al., 2008). Several studies have focused on the neural mechanisms that underlie the effects of MVF. Functional imaging studies have shown that the neural network related to MVF was attributed to the M1 and other motor-related areas such as premotor cortex and posterior superior temporal sulcus (Giraux and Sirigu, 2003; Sasaki et al., 2012). Nojima et al. (2012, 2015) found that motor-skill training using MVF rather than action observation induced the facilitation of motor cortical excitability in the M1 contralateral to the untrained hand. Recently, it was reported that self-paced sequential finger tapping related MVF increased hand speed as a factor of bradykinesia and the motor cortical excitability in PD patients (Bonassi et al., 2016). Thus, MVF intervention targeting the most clinically affected (severe) untrained side may have a beneficial effect on the acquisition of new motor skills related to increased motor cortical excitability in PD patients.

Non-invasive brain stimulation techniques have been shown to modulate brain processing and thereby influence behavior (Di Pino et al., 2014). The application of anodal tDCS over cortex can facilitate the excitability and anodal tDCS in combination with various kinds of motor training results in excitability changes in the human sensory and motor cortices (Nitsche and Paulus, 2000, 2011; Nitsche et al., 2003; Grundmann et al., 2011; Nitsche, 2011). It has also been reported that anodal tDCS enhances neuroplasticity and learning in older individuals and patients with stroke (Kang et al., 2016). Recently, in both aged and younger healthy subjects, motor skill training using MVF combined with anodal tDCS improved the dexterity of the untrained hand, compared to sham stimulation (Hoff et al., 2015; von Rein et al., 2015). Transcranial Magnetic Stimulation (TMS) is another technique of non-invasive brain stimulation and provide information on the conductivity of corticospinal neurons and the excitatory and inhibitory systems in the primary motor cortex (Cantello et al., 1991, 1992; Ridding and Rothwell, 1997).

Based on these studies, it is not yet known whether motor skill training using MVF is useful to patients with PD in order to improve untrained hand performance dependent on the time course of training; and whether MVF combined with anodal tDCS over M1 causes an additional effect based on increased motor cortical plasticity in PD. To elucidate this, we firstly applied motor skill training using MVF (intervention) to patients with PD and age matched healthy subjects. Then we secondly applied intervention combined with tDCS over M1 contralateral to the untrained hand in patients with PD.

## Materials and Methods

### Subjects

Eighteen patients with PD (mean age ± SD: 70.6 ± 5.4 years, 8 male and 10 female) and 10 age-matched healthy subjects (HS) (mean age ± SD: 68.1 ± 5.6 years, 7 male and 3 female) participated in the study. All of the patients fulfilled the United Kingdom Brain Bank Criteria (Hughes et al., 1992) and corresponded to categories 2 or 3 of the Modified Hoehn and Yahr Scale in the off-medication state. In order to control hand laterality, we recruited right-handed subjects according to the Edinburgh Inventory (Oldfield, 1971). In addition, in all PD patients the left side was the most affected (Table 1); as they performed the motor-skill training using MVF, the better performance of the right hand was used. Therefore, it was needed for them to rotate the ball more than twice especially on the right side. Moreover, even though in their affected left side, since we evaluated the number of the ball rotation, we excluded the patients who were not able to rotate the ball at all. All patients were evaluated after being off-medication (levodopa replacement) for 12 h. The motor function of the patients in the off-medication state was assessed in accordance with the motor section of the Unified Parkinson’s Disease Rating Scale (UPDRS). The clinical subtype of akinesic-rigid and tremor dominant were diagnosed based on previous paper by two neurologists (Lewis et al., 2005). The Mini-Mental State Examination (MMSE) and the Frontal Assessment Battery (FAB) (Dubois et al., 2000) were also performed. Attention and fatigue were assessed with the visual analog scale questionnaire (Attention scale, 1–7: 1, no attention; 7, highest level of attention) and the Chalder fatigue scale (Chalder et al., 1993) after the experiment on both days. All of the subjects provided written informed consent according to the dictates of the Nagoya City University Hospital Trust Ethics Committee (protocol number 46-13-0004). The experimental procedure was conformed to the Ethics of the World Medical Association (Declaration of Helsinki) and was approved by the university hospital medical information network in Japan. All methods were carried out in accordance with relevant guidelines and regulations.

**Table 1:** Clinical profile of patients with Parkinson’s disease.

Patient	Age (years)	Gender	Duration (years)	Condition	Affected side	Yahr	UPDRS part 3	UPDRS (item 20–25) Affected/unaffected side	Subtype	LEDD (mg/days)
1	76	F	4	Real	Left	2	19	8/3	Tremor	200
2	64	M	3	Real	Left	3	26	8/4	Akinesic-rigid	200
3	74	F	4	Real	Left	2	12	3/2	Akinesic-rigid	175
4	68	M	5	Real	Left	2	19	5/3	Tremor	300
5	69	F	8	Real	Left	3	9	6/3	Akinesic-rigid	350
6	78	F	11	Real	Left	3	7	4/0	Akinesic-rigid	559
7	69	F	8	Real	left	3	9	6/3	Akinesic-rigid	350
8	68	F	2	Real	left	2	6	4/2	Akinesic-rigid	200
9	76	F	13	Real	left	3	10	3/1	Akinesic-rigid	582
10	78	M	8	Sham	left	3	28	8/4	Tremor	100
11	68	F	10	Sham	left	3	25	7/6	Akinesic-rigid	75
12	69	F	7	Sham	left	3	23	5/4	Akinesic-rigid	125
13	70	F	1	Sham	left	3	9	3/2	Tremor	200
14	74	M	4	sham	left	3	19	8/3	Akinesic-rigid	300
15	69	F	9	Sham	left	3	9	4/2	Akinesic-rigid	674
16	72	M	2	Sham	left	2	6	3/0	Akinesic-rigid	250
17	60	M	4	Sham	left	3	17	7/3	Akinesic-rigid	50
18	76	M	13	Sham	left	3	18	6/5	Akinesic-rigid	490


*Yahr: Hoehn and Yahr, UPDRS: Unified Parkinson’s Disease Rating Scale.*

### Experimental Procedure

**FIGURE 1:**
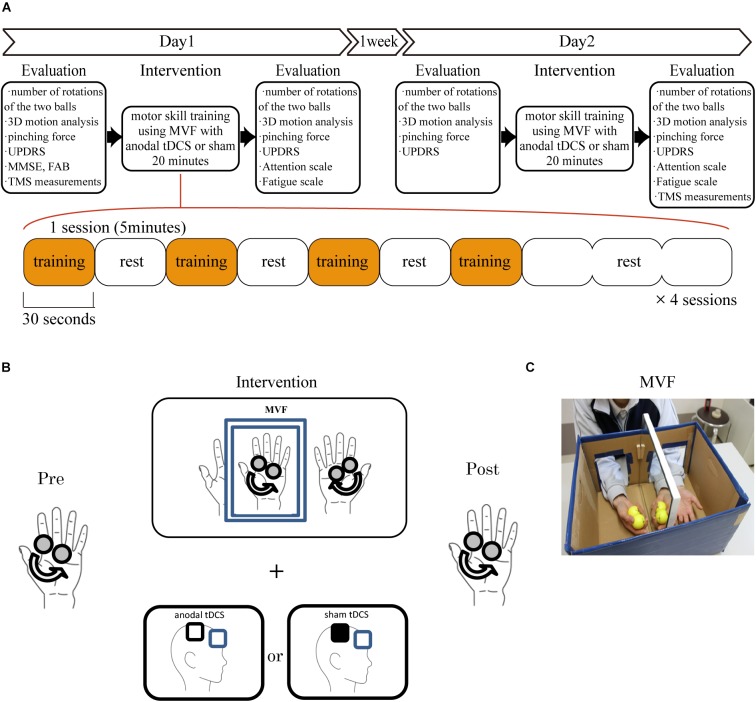
Experimental procedure. **(A)** The motor skill training was performed twice on two separate days (days 1 and 2) 1 week apart. The subjects underwent intervention over the right M1 for 20 min on each day. They performed four sessions of the behavioral task which comprised four sets of motor training for 30 s and rest for 30 s between each trial and 90 s rest in final part. The behavioral evaluation sessions were performed before and after the intervention on each day. The TMS evaluation was performed before day 1 and after intervention on day 2. **(B)** The ball-rotation task involved rotating two cork balls as fast as possible in a counterclockwise direction using the right hand. In PD, subjects were randomly divided the groups with tDCS or sham stimulation over the right M1. **(C)** For motor-skill training using MVF, the subjects placed both hands inside a box made of paper and mirrored glass, and performed the above behavioral task using their right hand.

All HS were recruited to a sham stimulation group. Although it should be interesting to look for any tDCS-induced change also in HS, the previous paper had already reported the effect of tDCS to the motor skill learning related to the same ball rotation task using mirror visual feedback in HS (Hoff et al., 2015; von Rein et al., 2015). Therefore, we plan the protocol of the real tDCS stimulation only to PD. In PD, eighteen patients were randomly divided to tDCS (mean age ± SD: 70.4 ± 6.0 years, 3 male and 6 female) and sham stimulation groups (mean age ± SD: 70.8 ± 5.0 years, 5 male and 4 female). The experimenter who do not perform the intervention and evaluation randomly assigned patient using a random number by Excel software. Since Nojima et al. (2012) reported that the number of ball rotations of left hand increased significantly after the intervention of mirror condition but non-mirror group in HS, we did not apply the non-mirror condition to the PD in this study. To elucidate the effect of motor skill training using MVF dependent on the time course, the intervention was performed on two separate days (day 1 and day 2), 1 week apart (Figure 1A). The intervention included four sessions of motor-skill training using MVF for 20 min which was comprised of four sets of training for 30 s each with 30 s of rest between each trial and 90 s rest in final part (Figure 1A). Since the intervention consisted of four sessions of motor-skill training using MVF for 20 min, patients were applied either tDCS or sham stimulation during total time of intervention (Figure 1B,C).

The behavior evaluation session was performed before (pre) and immediately after (post) intervention on both days (pre day 1, post day 1, pre day 2, and post day 2). In the evaluation session, the motor performance of the left hand was scored using the number of rotations of the two balls performed over 30 s, recorded throughout the evaluation session using a video camera and analyzed offline by a researcher. The mean maximal pinching force (PF) between the index finger and the thumb at five times the pinch gauge (in kgf) and the UPDRS (motor) score were also assessed. Attention and fatigue were assessed after each day (post day 1 and post day 2). The transcranial magnetic stimulation (TMS) measurements included the mean motor-evoked potential (MEP) amplitudes, input-output (I-O) function, and the mean silent period (SP) of the left APB, recorded twice (pre day 1 and post day 2). It took much time when TMS measurements were performed in four time points in PD, which may affect the behavioral data because of sever fatigue. Moreover, our main purpose is to clarify whether this total intervention can produce the change of motor cortical systems in PD. Therefore, we evaluated subject’s cortical excitatory and inhibitory systems by means of TMS only two time points. All of the data were stored on a computer, and a researcher blind to subject type analyzed behavioral data.

### Behavioral Task and Motor Skill Training Using MVF

The target task was using the right hand to rotate two cork balls (diameter, 30 mm) as fast as possible in a counterclockwise direction (Figure 1A,B). For motor skill training using MVF, the subjects were instructed to place both of their hands inside a box made of paper and mirrored glass that prevented the direct view of the right hand but allowed an indirect view via the mirror (Figure 1B). The subjects were instructed to observe the movements of the right hand in a mirror that provided MVF of their performance in the ball rotation task. This behavioral task and motor skill training using MVF were originally developed by Nojima et al. (2012).

### Transcranial Magnetic Stimulation

Each subject was seated in an armchair with arms placed on the armrest. Surface electromyograms (EMGs) were recorded from the left APB muscle with a pair of silver electrodes. The EMGs were amplified and filtered, and digitized at a sampling rate of 10 kHz using the Labview system. The position of the EMGs was marked on the hand and recorded by video camera. The TMS was produced using a 7-cm figure-of-eight coil connected to a Magstim^®^ 200 Monophasic Transcranial Stimulator (The Magstim Co., Whitland, Dyfed, United Kingdom). At the beginning of each day the optimal motor points for APB were determined following a standard procedure (Rossini et al., 1994). The optimal motor points for eliciting the best motor response from the left APB muscle with the coil held ∼45° to the midsagittal line were established over the right M1, which was determined in 5 mm steps around the presumed motor hand area. The current of TMS was induced by posterior-anterior direction. The determined coil position was marked on the scalp and its coordinates on midsagittal (nasion-inion line) and biauricular (line connecting external auditory meati) axes in relationship to the vertex were recorded according to the previous repetitive TMS study (Filipović et al., 2010). This recorded coil position was also used for the recording in post day 2. The resting motor threshold was defined on pre day 1, in accordance with a previous study, as the lowest stimulus intensity required to elicit MEP with a peak-to-peak amplitude of >50 μV in the left APB muscle in at least five out of 10 trials (Rossini et al., 1994).

In order to assess corticospinal excitability, MEP amplitudes were measured with the fixed stimulus intensity of the TMS machine adjusted to 120% of resting motor threshold at the target APB muscle in pre day 1. The mean peak-to-peak MEP amplitudes for 10 trials were measured. The recruitment of the corticospinal projection (I-O function) from the right M1 was also measured (Ridding and Rothwell, 1997). The intensities of single TMS stimuli were individually adapted according to the resting motor threshold to evaluate the I-O function. Ten MEPs were recorded from the left APB muscle at intensities of 50, 80, 90, 100, 110, 120, and 150% of the resting motor threshold and averaged at each intensity.

The SP was also measured (10 replicates) to assess the motor inhibitory system. We evaluated the SP by applying TMS to the right M1 at 140% of the resting motor threshold during low-force activation of the APB (Fuhr, 1991; Ziemann et al., 1993; Kojima et al., 2013). Subjects maintained a voluntary isometric contraction at approximately 20% of their maximum voluntary contraction by providing feedback from the surface EMG on a computer screen. The duration of ten SP was measured from the end of the MEP until the restart of a constant EMG activity. We employed a method by which the amplitude and onset were measured automatically to minimize observer bias using a custom-made MATLAB program (MathWorks, Natick, MA, United States).

We had also confirmed the hot spot by using TMS just before the intervention of day 2. Moreover, the coil position was measured on the scalp in accordance with midsagittal (nasion-inion line) and biauricular (line connecting external auditory meati) axes relative to the vertex on day 1 and was adjusted before the intervention of day 2.

In PD, during the TMS experiment, EMG background activities were continuously observed online; when rigidity or tremor prominently appeared, the examiner left as long as patients needed to relax throughout the experiment and started recording when the background EMG was silent.

### Transcranial Direct Current Stimulation

A weak direct current of 2 mA was delivered via saline-soaked sponge electrodes using a DC Stimulator Plus (neuroConn GmbH, Ilmenau, Germany). TMS was used to identify the functional landmark of the right M1 as the optimal position. The current density at the stimulation electrodes was 0.025 mA/cm^2^ in accordance with safety criteria and far below the threshold for tissue damage (Nitsche, 2003). Either anodal tDCS or sham stimulation was applied to the right M1 during the intervention on both day 1 and day 2. The impedance of the stimulation electrodes was kept below 10 kΩ. In all of the conditions, the stimulating electrode was placed above the right M1, while the reference electrode of the cathode was placed above the frontal orbit. During tDCS, the current was increased at the beginning and decreased at the end of the protocol (20 min) over 30 s in a ramp-like manner, on the basis of previous reports (Nitsche et al., 2003). In the sham condition, the current was applied for only 30 s. This protocol has been demonstrated to reliably blind subjects with respect to the stimulation condition (Gandiga et al., 2006).

### Behavioral Data Analysis

For evaluation of motor performance, the dexterity of the left hand was examined by counting the number of two-ball rotations during 30 s. Two experimenters separately counted the number of two balls rotation, one of which was marked by color, during 30 s by using Video monitor on off-line and adjusted each number. In addition, for monitoring acceleration of the left thumb, a three-dimensional motion analysis system (Locus 3D MA-3000, Anima Corp., Tokyo, Japan) was used with four infrared cameras to capture and analyze motion with a sampling frequency of 100 Hz (Yamaguchi et al., 2015). Reflective markers of 5-mm diameter were attached on the first joint of the left thumb. The mean peak acceleration of the thumb movements during two-ball rotations during 30 s was calculated and expressed in cm/s^2^.

### Statistical Analysis

To determine the effect of motor skill training using MVF dependent on time course in patients with PD, we compared the outcome in behavior and TMS parameters between HS and PD-sham. The number of ball rotations, peak acceleration and PF were used as the behavioral variables, while the mean MEP amplitude, I-O function and SP from the TMS were used as the physiological variables. The effect of these variables was evaluated using a two-way repeated measures analysis of variance (RM-ANOVA), with group (PD, HS) as a between-subjects factor and time (pre day 1, post day 1, pre day 2, and post day 2) as behavioral variables or pre day 1 and post day 2 as physiological variables. Attention and fatigue were also evaluated using RM-ANOVA. The Greenhouse-Geisser method was used to correct for non-sphericity. If the effect was significant, a *post hoc* *t*-test was performed on the data. The Bonferroni correction for multiple comparisons was also used where necessary.

To determine whether the intervention combined with anodal tDCS over the right M1 contributes to motor performance and facilitation in the M1 in patients with PD, the number of ball rotations, peak acceleration, PF and UPDRS motor score were used as the behavioral variables, while the mean MEP amplitude, I-O function and SP from the TMS were used as the physiological variables. The effect of these variables was evaluated using a two-way repeated measures analysis of variance (RM-ANOVA), with condition (sham, tDCS) as a between-subjects factor and time (pre day 1, post day 1, pre day 2, and post day 2) as behavioral variables or pre day 1 and post day 2 as physiological variables. The Greenhouse-Geisser method was used to correct for non-sphericity. If the effect was significant, a *post hoc* *t*-test was performed on the data. The Bonferroni correction for multiple comparisons was also used where necessary.

Results with *P*-values less than 0.05 were considered significant. All of the statistical analyses were performed with SPSS version 25.0 for Windows (IBM Japan, Tokyo, Japan).

## Results

Cognitive scores, measured by mini mental scale examination (MMSE) were 28.6 ± 1.7 in HS, 29.0 ± 1.4 in PD; frontal assessment battery (FAB) was 14.9 ± 2.2 in HS and 15.9 ± 1.5 in PD. The characteristics of PD patients are summarized in Table 1.

### The Effect of MVF on Behavior: HS vs. PD

**FIGURE 2:**
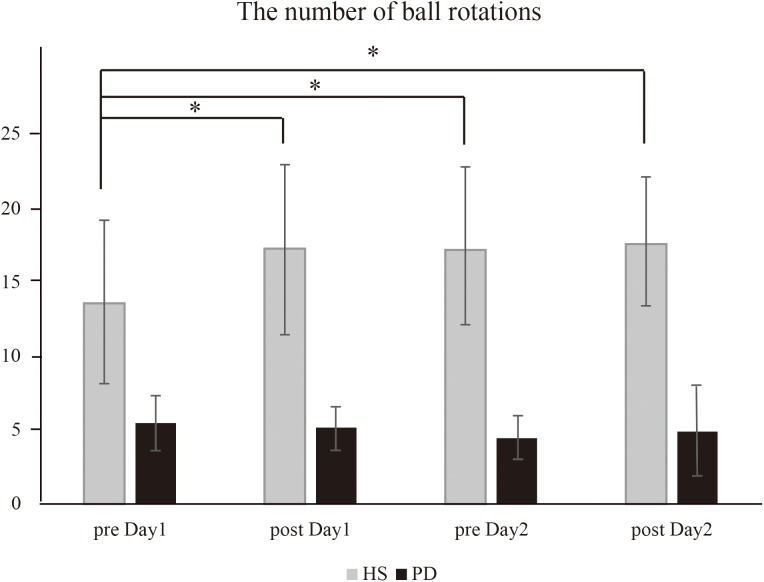
The number of ball rotations (HS vs. PD). In HS, the intervention caused a significant increase in the number of ball rotations on post day 1 (^∗^*P* = 0.003), pre day 2 (^∗^*P* = 0.001), and post day 2 (^∗^*P* = 0.01) compared to that on pre day 1. In contrast, in PD, the intervention caused no significant increase in the number of ball rotations.

Regarding the result of the number of two-ball rotations (Figure 2), there was a significant effect of group [^∗^*P* = 0.001, *F*(1,16) = 25.3] and time × group interaction [^∗^*P* = 0.002, *F*(3,48) = 5.88]. Thus, further analysis was performed on each group. In HS, *post hoc* analysis demonstrated the number of ball rotations that could be performed on post day 1, pre day 2, and post day 2 was significantly increased compared to those performed on pre day 1 (^∗^*P* = 0.003 on post day 1, ^∗^*P* = 0.001 on pre day 2, and ^∗^*P* = 0.01 on post day 2). In contrast, there was no significant additional improvement in the number of ball rotations performed between post day 1 and pre day 2 (*P* = 0.88), or between pre day 2 and post day 2 (*P* = 0.76). On the other hand, in PD, *post hoc* analysis demonstrated no significant difference in the number of ball rotations between any time points (*P* > 0.05).

The result of the mean peak acceleration in HS and PD are summarized as Table 2. There was a significant effect of group [^∗^*P* = 0.001, *F*(1,16) = 28.6] and time × group interaction [^∗^*P* = 0.001, *F*(1.9,30.4) = 9.59]. In HS, *post hoc* analysis demonstrated there were significantly increased peak accelerations between pre day 1 and pre day 2 or post day 2 (^∗^*P* = 0.01 on pre day 2 and ^∗^*P* = 0.008 on post day 2) and between pre day 2 and post day 2 (^∗^*P* = 0.02). In contrast, there was no significant additional improvement in the accelerations performed between pre day 1 and post day 1 (*P* = 0.09) and pre day 2 (*P* = 0.76). On the other hand, in PD, *post hoc* analysis demonstrated no significant difference in the accelerations between any time points (*P* > 0.05).

**Table 2:** Mean peak acceleration in HS and PD.

	Mean peak acceleration (cm/s^2^)			
	Pre day 1	Post day 1	Pre day 2	Post day 2
HS	60.9 ± 11.6	74.6 ± 29.7	76.3 ± 18.9^#^	96.5 ± 36.9^∗^,^$^
PD	38.2 ± 16.5	35.1 ± 15.5	32.8 ± 12.1	31.7 ± 15.9


*^#^*P* = 0.01 comparison to pre day 1, ^∗^*P* = 0.008 comparison to pre day 1, ^$^*P* = 0.02 comparison to pre day 2 (*post hoc* analysis). The values are in means ± standard deviations (SD).*

The result of the PF in HS and PD are summarized as Table 3. There was no significant effect of group (*P* = 0.16) or time × group interaction (*P* = 0.27).

**Table 3:** Mean maximal pinching force (in kgf) in HS and PD.

	Mean maximal pinching force (in kgf)			
	Pre day 1	Post day 1	Pre day 2	Post day 2
HS	7.4 ± 2.3	8.0 ± 2.4	8.1 ± 2.4	8.1 ± 2.4
PD	6.9 ± 1.4	7.5 ± 1.6	7.4 ± 1.9	7.4 ± 1.8


*The values are in means ± standard deviations (SD).*

The result of the attention and fatigue scores in HS and PD are summarized as Table 4. For attention, there was a significant main effect of group [^∗^*P* = 0.03, *F*(1,16) = 5.49] but no group × time interaction effect [*P* = 0.41, *F*(1,16) = 0.71]. For fatigue, there was no significant main effect of group [*P* = 0.21, *F*(1,16) = 1.73]; however, there was a group × time interaction effect [^∗^*P* = 0.002, *F*(1,16) = 13.6]. In PD, *post hoc* analysis demonstrated there was significantly increased fatigue score on post day 2 compared with post day 1 (^∗^*P* = 0.01). By contrast, in HS there was no significant change (*P* = 0.20).

**Table 4:** Attention and fatigue scores in HS and PD.

	Attention score		Fatigue score	
	
	Post day 1	Post day 2	Post day 1	Post day 2
HS	6.6 ± 1.0	6.3 ± 0.7	10.3 ± 2.4	11.5 ± 1.0
PD	5.8 ± 0.7	5.8 ± 0.4	14.7 ± 3.6	10.8 ± 5.2^∗^


*^∗^*P* = 0.01 comparison to post day 1 (*post hoc* analysis). The values are in means ± standard deviations (SD).*

### The Effect of MVF on Motor Cortical Excitatory and Inhibitory Systems: HS vs. PD

The resting motor thresholds for the left APB in HS were 54.8 ± 11.2% (pre day 1) and 50.8 ± 12.2% (post day 2) of the maximal stimulator output. The resting motor thresholds in PD patients were 57 ± 10.3% (pre day 1) and 57.1 ± 10.3% (post day 2). There were no significant effects of group (*P* = 0.19) or group × time interactions (*P* = 0.07).

**FIGURE 3:**
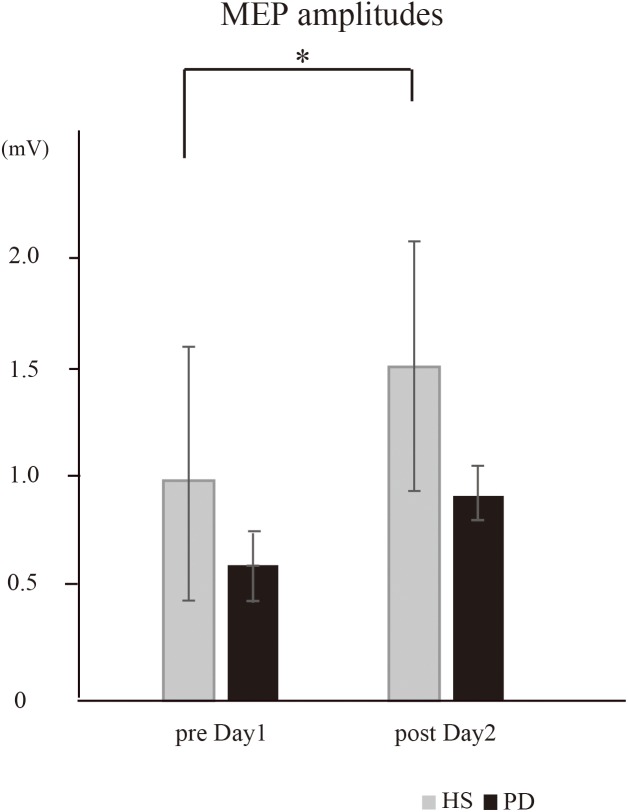
The MEP amplitudes recorded from the right APB (HS vs. PD). In HS, the intervention caused a significant increase in the mean MEP amplitudes on post day 2 compared to those on pre day 1 (^∗^*P* = 0.03). In PD, the intervention caused no significant increase in the mean MEP amplitudes (*P* = 0.12).

The effect of the intervention on the motor cortical excitatory system was evaluated by the changes in MEP amplitude recorded from the left APB between HS and PD. There was a significant effect of group [^∗^*P* = 0.04, *F*(1,16) = 4.55] and time × group interaction [^∗^*P* = 0.05, *F*(1,16) = 3.8]. Thus, further analysis was performed in each group. In HS, *post hoc* analysis demonstrated the MEP amplitude on post day 2 was significantly increased compared to that in pre day 1 (^∗^*P* = 0.03); in contrast, MEP amplitude in PD was not affected (*P* = 0.12) (Figure 3).

**FIGURE 4:**
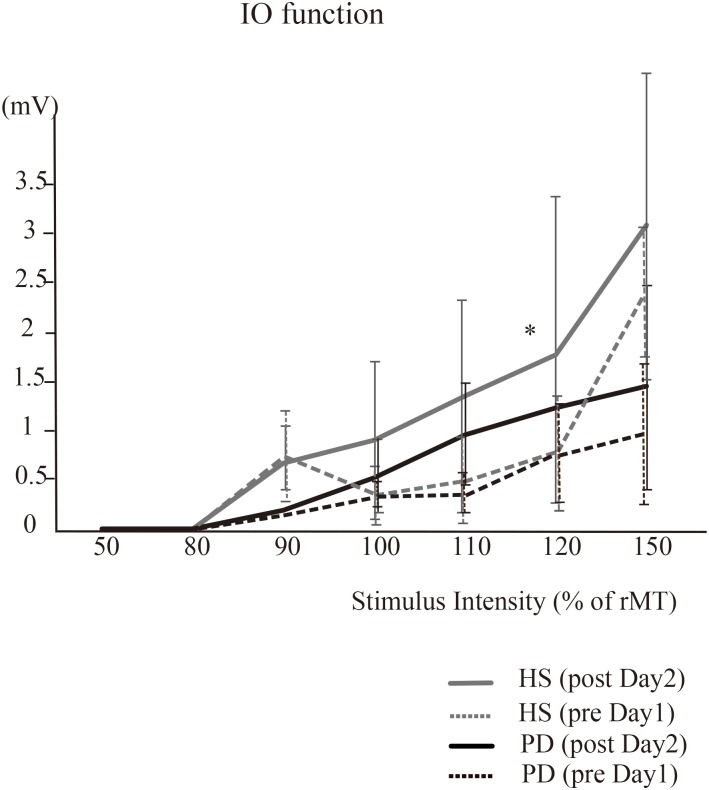
The IO function (HS vs. PD). For the I-O function in HS, MEP amplitudes at the intensity of 120% on post day 2 were significantly increased compared to those performed on pre day 1 (^∗^*P* = 0.04). In PD, there were no significant differences in the MEPs. At 50, 80, 90, 100, and 150% intensity, there were no significant effects of time or time × group interaction in either HS or PD.

For the I-O function of MEP amplitudes at an intensity of 120%, there was a significant effect of group [^∗^*P* = 0.00, *F*(1,16) = 28.5] and time × group interaction [^∗^*P* = 0.04, *F*(1,16) = 4.51]. In HS, *post hoc* analysis demonstrated that the MEPs on post day 2 were significantly increased compared to those on pre day 1 (^∗^*P* = 0.04) (Figure 4). On the other hand, in PD, *post hoc* analysis demonstrated no significant difference in the MEPs (*P* > 0.05). At 50, 80, 90, 100, and 150% intensity, there were no significant effects of time or time × group interaction.

The effect of the intervention on motor cortical inhibitory system was evaluated by the changes in the SP between HS and PD. The SP were 92.5 ± 16 and 85.9 ± 20.3 (pre day 1), and 88.3 ± 16.1 and 87.6 ± 22.4 (post day 2) in the HS and PD groups, respectively. There was no effect of group (*P* = 0.69) or time × group interaction (*P* = 0.34).

### The Effect of the Intervention (MVF+tDCS) on Behavior and Motor Cortical Excitability: PD-tDCS vs. PD-Sham

**FIGURE 5:**
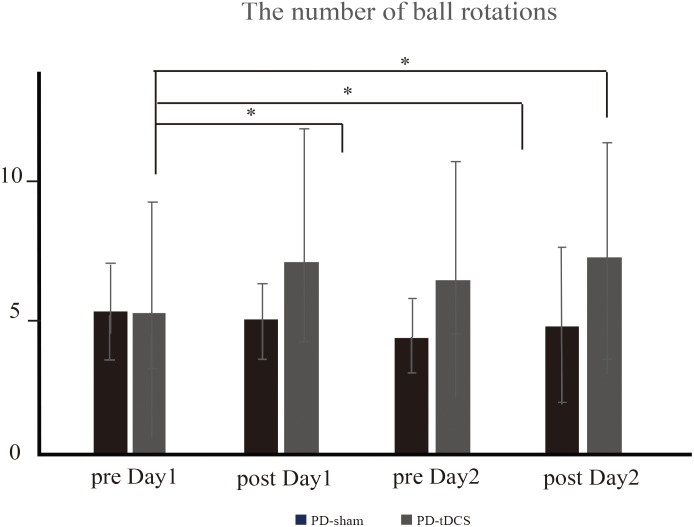
The number of ball rotations (PD-tDCS vs. PD sham). In PD-tDCS, the intervention caused a significant increase in the number of ball rotations on post day 1 (^∗^*P* = 0.006), pre day 2 (^∗^*P* = 0.006), and post day 2 (^∗^*P* = 0.02) compared to that on pre day 1.

Since the number of ball rotations, peak acceleration and motor cortical excitatory system were significantly impaired in PD, we evaluated the effect of tDCS over the right M1 on these factors. For the number of two-ball rotations, there was an effect of condition [^∗^*P* = 0.05, *F*(1,15) = 4.12] but no time × condition interaction effect [*P* = 0.17, *F*(3,45) = 1.73]. Thus, further analysis was performed in the tDCS condition. The *post hoc* analysis revealed that the intervention + tDCS led to a significant increase in the number of ball rotations on post day 1 and pre day 2 compared to that on pre day 1 (post day 1, ^∗^*P* = 0.006; pre day 2, ^∗^*P* = 0.006; post day 2, ^∗^*P* = 0.02) (Figure 5). However, the intervention did not cause a further increase in the number of rotations between post day 1 and pre day 2 (*P* = 0.05) or between pre day 2 and post day 2 (*P* = 0.25). There was no significant effect of condition (*P* = 0.9) or time × condition interaction (*P* = 0.31) on the mean peak acceleration.

The UPDRS scores were 18.9 ± 7.4 (pre day 1), 18.1 ± 6.3 (post day 1), 18.9 ± 7.4 (pre day 2), and 17.3 ± 7.0 (post day 2) in the PD-sham group, and 15.3 ± 7.2 (pre day 1), 14.5 ± 5.9 (post day 1), 13.8 ± 4.7 (pre day 2), and 13.3 ± 5.9 (post day 2) in the PD-tDCS group. There were no significant main effects of time [*P* = 0.25, *F*(1.29,12.94) = 1.49] or condition × time interaction [*P* = 0.51, *F*(1.29,12.94) = 0.55] on the UPDRS score.

The resting motor thresholds in PD-tDCS were 55.7 ± 10.8% (pre day 1) and 54.6 ± 9.8% (post day 2). Regarding motor cortical excitability, the mean MEP amplitudes in the PD-tDCS group were 0.79 ± 0.3 mV in pre day 1 and 0.83 ± 0.4 mV in post day 2. There were no significant effects of condition (*P* = 0.11) or time × condition interaction (*P* = 0.82).

For the I-O function of MEP amplitudes, at an intensity of 150%, there were significant effects of time [*F*(1,15) = 4.16, *P* = 0.05] and condition [*F*(1,15) = 5.29, *P* = 0.04], but no time × condition interaction effect [F(1,15) = 2.8, *P* = 0.1]. The *post hoc* analysis demonstrated that the MEPs on post day 2 were significantly increased compared to those performed on pre day 1 (^∗^*P* = 0.04). At 50, 80, 90, 100, and 120% intensity, there were no significant effects of time or time × condition interaction.

## Discussion

This study revealed that behavioral improvement and motor cortical plasticity caused by motor skill training using MVF were impaired in patients with PD compared to HS; improvements of behavior and motor cortical excitability occurred if anodal tDCS was applied to the right M1 contralateral to the untrained left hand. These findings suggest that the combination of MVF and tDCS could be a promising strategy to improve motor skills in a specific manner dependent on the motor excitatory system.

In HS, the number of ball rotations and the acceleration of the left thumb were significantly increased through repeated motor skill training using MVF both on day 1 and day 2, depending on the facilitated MEP amplitude and I-O function of 120% intensity. These results in HS were compatible with those of previous studies which also demonstrated improved hand dexterity after motor skill training using MVF related to inducing motor cortical plasticity in contralateral M1(Hoff et al., 2015; von Rein et al., 2015). The ball rotation task skill using MVF was acquired on day 1 and was retained until 1 week after the initial training (day 2); however, additional behavioral improvement did not occur on day 2. This behavioral change was likely mediated by an improved dexterity of the left hand as opposed to a change in PF, indicating that the task-specific manner of behavioral improvement depends on the motor excitatory system not inhibitory system. The motor cortical neurons of the M1 are reported to encode specific movement (Muir and Lemon, 1983; Rizzolatti et al., 1996a,b; Kakei et al., 2001). Our results suggest that the encoding of a new motor memory such as ball rotation, although in the form of mirrored movement, can be improved by the enhanced excitability or synaptic efficacy of the appropriate neuronal population in the M1.

On the other hand, in PD, the number of ball rotations and the facilitation of the MEP amplitude did not improve through repeated skill training on day 1 and day 2. Our result suggest that even though combined with the fine visual input the motor skill acquisition did not occur and motor cortical excitability did not changed in PD. Most studies of PD report impaired acquisition in serial reaction time task paradigms, with a relatively preserved early stage of skill acquisition and impaired retention of short and long-term motor memories in adaptation tasks (Agostino et al., 1996; Jessop et al., 2006; Muslimovic et al., 2007; Pendt et al., 2011). Recently, we reported that the repetitive skill training did not result in effective improvement of motor performance in PD, which is related to reduce dopamine release in the contralateral putamen by using ^11^C-raclopride positron emission tomography (Kawashima et al., 2018). In previous studies of PD using motor imagery tasks, a TMS study reported that motor cortical excitability in the M1 was impaired during action imagination of the hand; a task-based fMRI study reported the activated brain areas including (BG)-thalamocortical circuitry were altered (Thobois et al., 2000; Tremblay et al., 2008). Moreover, the study of an animal model of PD showed that plasticity in the motor cortex is important for the acquisition and that dopamine depletion resulted in structural changes in the motor cortex and atypical synaptic adaptations (Molina-Luna et al., 2009; Guo et al., 2015). Based on these findings, in PD, the impaired facilitation of the MEP amplitude during the motor skill training using MVF may be caused by dopamine depletion and secondary alteration of modulation in BG-thalamocortical circuitry.

In order to increase the facilitation effect of motor cortical excitability for improving affected hand dexterity in PD, we applied tDCS over the contralateral M1 during motor skill training using MVF. When the tDCS was applied to the right M1 in PD, the number of ball rotations using the untrained left hand were significantly increased on day 1 and the effect was retained after 1 week. Although the MEP amplitude recorded by 120% of resting motor threshold was not increased, I-O function at 150% intensity was significantly increased after day 2. It is well known that application of anodal tDCS in combination with various kinds of motor training results in excitability changes and induction of homeostatic plasticity in the human sensory and motor cortices (Nitsche and Paulus, 2000, 2011, Nitsche et al., 2003, Grundmann et al., 2011; Nitsche, 2011). Moreover, many studies in PD examining the effects of tDCS over the M1, premotor, prefrontal, Cz area, etc., have reported an effect on the change in UPDRS (motor) scores, gait speed, working memory, etc., compared to sham stimulation (Fregni et al., 2006; Benninger et al., 2010; Kaski et al., 2014). Anodal tDCS over the M1 is known to alter the resting membrane potentials of M1 neurons, leading to an increase in cortical excitability that has been proposed to help in the compensation of the reduced BG thalamo-cortical drive (Fregni et al., 2006; Benninger et al., 2010). In this study, the behavioral improvement occurred with facilitated I-O function, which may be caused by the altered excitability of the M1.

Reis et al. (2008) reported beneficial effects of tDCS in combination with motor training, and it is reasonable to assume that such neuroplasticity-based interventions might constitute a useful rehabilitative tool for PD patients. On the other hand, a recent clinical review reported that there was insufficient evidence to determine the effects of tDCS in reducing off time and on time with dyskinesia and for improving the health-related quality of life, disability, and impairment in patients with PD (Elsner et al., 2016). In the present study, the UPDRS (motor) score was not changed by repeated intervention with tDCS over the M1. However, we applied the interventions 1 week apart in this study. Short- and long-term motor skill training typically results in functional brain alterations in a variety of motor-related brain regions, including the M1 (Doyon and Benali, 2005; Doyon, 2008). Moreover, a previous study provides evidence that repeated application of non-invasive brain stimulation over multiple days might even prolong such behavioral effects (Reis et al., 2009). Taken together this evidence could be combined to develop new effective rehabilitation.

In the present study, the motor training time was not so long, since the intervention consisted of 30 s of rest between each trial. However, PD showed significantly increased fatigue score on post day 2 compared with post day 1. A progressive slowing in speed or progressive decrease in the amplitude of repetitive movements are observed in patients with PD, which is known as the sequence effect (Agostino et al., 1992, 1994). This performance decline has also been observed in drug-naïve patients with PD during finger tapping and the repetitive movements involved in a pegboard task (Kang et al., 2010). Although the related pathogenesis is still unclear, it may be caused by freezing rather than fatigue. Considering these findings, the behavioral improvement by tDCS in PD may be explained mostly by the effect on acquired motor skill with MVF not on fatigue.

There are several limitations to our study. Because the sample size was as small as nine cases in each group, a significant difference between tDCS and sham stimulation in PD may not have been detected in TMS parameters other than the I-O curve. In the protocol of this study, we did not apply the intervention of MVF alone without sham stimulation to the PD patients. In PD, it is well known the placebo effect on the improvement of motor behavior and it should be carefully assessed in clinical trials (Frisaldi et al., 2017). In the previous study of sequential finger tapping with MVF, MVF training increased movement speed in untrained hand (Bonassi et al., 2016). Since our result showed no increase of the number of ball rotation in untrained hand by the intervention of MVF with sham stimulation, the involvement of placebo effect seems to be less likely.

Although we applied the interventions 1 week apart in this study, repeated application of tDCS over sequential days might even prolong such behavioral effects and motor cortical plasticity in PD. Further studies are needed to determine how to maximize the beneficial effect of tDCS before this method can be applied to patients with PD.

## Ethics Statement

All of the subjects provided written informed consent according to the dictates of the Nagoya City University Hospital Trust Ethics Committee (protocol number 46-13-0004). The experimental procedure conformed to the Ethics of the World Medical Association (Declaration of Helsinki) and was approved by the university hospital medical information network in Japan. All methods were carried out in accordance with relevant guidelines and regulations.

## Author Contributions

MH executed the research and statistical analysis and wrote the first draft of the manuscript. YU and IN organized and executed the research and statistical analysis and reviewed and critiqued the manuscript. YS, KS, and SI executed the research. AS, GY, NM, and IW reviewed and critiqued the manuscript.

## Conflict of Interest Statement

The authors declare that the research was conducted in the absence of any commercial or financial relationships that could be construed as a potential conflict of interest.

## Abbreviations

APB: abductor pollicis brevis
tDCS: transcranial direct current stimulation

## References

[B1] Abbruzzese G. Avanzino L. Marchese R. Pelosin E. (2015). Action observation and motor imagery: innovative cognitive tools in the rehabilitation of Parkinson’s disease. *Parkinsons Dis.* 2015:124214 10.1155/2015/124214 PMC4606219 26495150

[B2] Agostino R. Berardelli A. Formica A. Accornero N. Manfredi M. (1992). Sequential arm movements in patients with Parkinson’s disease, huntington’s disease and dystonia. *Brain* 115 1481–1495. 10.1093/brain/115.5.1481 1422799

[B3] Agostino R. Berardelli A. Formica A. Stocchi F. Accornero N. Manfredi M. (1994). Analysis of repetitive and nonrepetitive sequential arm movements in patients with Parkinson’s disease. *Mov. Disord.* 9 311–314. 10.1002/mds.870090305 8041371

[B4] Agostino R. Sanes J. N. Hallett M. (1996). Motor skill learning in Parkinson’s disease. *J. Neurol. Sci.* 139 218–226. 10.1016/s0022-510x(96)00060-3 8856656

[B5] Altschuler E. L. Wisdom S. B. Stone L. Foster C. Galasko D. Llewellyn D. M. (1999). Rehabilitation of hemiparesis after stroke with a mirror. *Lancet* 353 2035–2036. 10.1016/s0140-6736(99)00920-4 10376620

[B6] Benninger D. H. Lomarev M. Lopez G. Wassermann E. M. Li X. Considine E. (2010). Transcranial direct current stimulation for the treatment of Parkinson’s disease. *J. Neurol. Neurosurg. Psychiatry* 81 1105–1111. 10.1136/jnnp.2009.202556 20870863 PMC4162743

[B7] Bonassi G. Pelosin E. Ogliastro C. Cerulli C. Abbruzzese G. Avanzino L. (2016). Mirror visual feedback to improve bradykinesia in Parkinson’s Disease. *Neural Plast.* 2016:8764238 10.1155/2016/8764238 PMC4983670 27563470

[B8] Cantello R. Gianelli M. Bettucci D. Civardi C. De Angelis M. S. Mutani R. (1991). Parkinson’s disease rigidity: magnetic motor evoked potentials in a small hand muscle. *Neurology* 41 1449–1456. 10.1212/wnl.41.9.1449 1891097

[B9] Cantello R. Gianelli M. Civardi C. Mutani R. (1992). Magnetic brain stimulation: the silent period after the motor evoked potential. *Neurology* 42 1951–1959. 10.1212/wnl.42.10.1951 1407578

[B10] Castiello U. Ansuini C. Bulgheroni M. Scaravilli T. Nicoletti R. (2009). Visuomotor priming effects in Parkinson’s disease patients depend on the match between the observed and the executed action. *Neuropsychologia* 47 835–842. 10.1016/j.neuropsychologia.2008.12.016 19138692

[B11] Chalder T. Berelowitz G. Pawlikowska T. Watts L. Wessely S. Wright D. (1993). Development of a fatigue scale. *J. Psychosom. Res.* 37 147–153. 8463991 10.1016/0022-3999(93)90081-p

[B12] DeLong M. R. (1990). Primate models of movement disorders of basal ganglia origin. *Trends Neurosci.* 13 281–285. 10.1016/0166-2236(90)90110-v 1695404

[B13] Di Pino G. Pellegrino G. Assenza G. Capone F. Ferreri F. Ranieri F. (2014). Modulation of brain plasticity in stroke: a novel model for neurorehabilitation. *Nat. Rev. Neurol.* 10 597–608. 10.1038/nrneurol.2014.162 25201238

[B14] Doyon J. (2008). Motor sequence learning and movement disorders. *Curr. Opin. Neurol.* 21 478–483. 10.1097/WCO.0b013e328304b6a3 18607210

[B15] Doyon J. Benali H. (2005). Reorganization and plasticity in the adult brain during learning of motor skills. *Curr. Opin. Neurobiol.* 15 161–167. 10.1016/j.conb.2005.03.004 15831397

[B16] Dubois B. Slachevsky A. Litvan I. Pillon B. (2000). The FAB a frontal assessment battery at bedside. *Neurology* 55 1621–1626. 10.1212/wnl.55.11.1621 11113214

[B17] Elsner B. Kugler J. Pohl M. Mehrholz J. (2016). Transcranial direct current stimulation (tDCS) for idiopathic Parkinson’s disease. *Cochrane Database Syst. Rev.* 7:CD010916 10.1002/14651858.CD010916.pub2 PMC6457946 27425786

[B18] Filipović S. R. Rothwell J. C. Bhatia K. (2010). Slow (1 Hz) repetitive transcranial magnetic stimulation (rTMS) induces a sustained change in cortical excitability in patients with Parkinson’s disease. *Clin. Neurophysiol.* 121 1129–1137. 10.1016/j.clinph.2010.01.031 20350836 PMC2997700

[B19] Fregni F. Boggio P. S. Santos M. C. Lima M. Vieira A. L. Rigonatti S. P. (2006). Noninvasive cortical stimulation with transcranial direct current stimulation in Parkinson’s disease. *Mov. Disord.* 21 1693–1702. 10.1002/mds.21012 16817194

[B20] Frisaldi E. Carlino E. Zibetti M. Barbiani D. Dematteis F. Lanotte M. (2017). The placebo effect on bradykinesia in Parkinson’s disease with and without prior drug conditioning. *Mov. Disord.* 32 1474–1478. 10.1002/mds.27142 28895186

[B21] Fuhr P. (1991). Spinal motor neuron excitability during the silent period after cortical stimulation. *Electroencephalogr. Clin. Neurophysiol.* 81 257–262. 10.1016/0168-5597(91)90011-l 1714819

[B22] Gandiga P. C. Hummel F. C. Cohen L. G. (2006). Transcranial DC stimulation: a tool for double-blind sham-controlled clinical studies in brain stimulation. *Clin. Neurophysiol.* 117 845–850. 10.1016/j.clinph.2005.12.003 16427357

[B23] Giraux P. Sirigu A. (2003). Illusory movements of the paralyzed limb restore motor cortex activity. *Neuroimage* 20(Suppl. 1), S107–S111. 14597303 10.1016/j.neuroimage.2003.09.024

[B24] Grundmann L. Rolke R. Nitsche M. A. Pavlakovic G. Happe S. Treede R. D. (2011). Effects of transcranial direct current stimulation of the primary sensory cortex on somatosensory perception. *Brain Stimul.* 4 253–260. 10.1016/j.brs.2010.12.002 22032740

[B25] Guo L. Xiong H. Kim J. I. Wu Y. W. Lalchandani R. R. Cui Y. (2015). Dynamic rewiring of neural circuits in the motor cortex in mouse models of Parkinson’s disease. *Nat. Neurosci.* 18 1299–1309. 10.1038/nn.4082 26237365 PMC4551606

[B26] Hoff M. Kaminski E. Rjosk V. Sehm B. Steele C. J. Villringer A. (2015). Augmenting mirror visual feedback-induced performance improvements in older adults. *Eur. J. Neurosci.* 41 1475–1483. 10.1111/ejn.12899 25912048

[B27] Hughes A. J. Daniel S. E. Kilford L. Lees A. J. (1992). Accuracy of clinical diagnosis of idiopathic Parkinson’s disease: a clinico-pathological study of 100 cases. *J. Neurol. Neurosurg. Psychiatry* 55 181–184. 10.1136/jnnp.55.3.181 1564476 PMC1014720

[B28] Jessop R. T. Horowicz C. Dibble L. E. (2006). Motor learning and Parkinson disease: refinement of movement velocity and endpoint excursion in a limits of stability balance task. *Neurorehabil. Neural Repair.* 20 459–467. 10.1177/1545968306287107 17082501

[B29] Kakei S. Hoffman D. S. Strick P. L. (2001). Direction of action is represented in the ventral premotor cortex. *Nat. Neurosci.* 4 1020–1025. 10.1038/nn726 11547338

[B30] Kang N. Summers J. J. Cauraugh J. H. (2016). Transcranial direct current stimulation facilitates motor learning post-stroke: a systematic review and meta-analysis. *J. Neurol. Neurosurg. Psychiatry* 87 345–355. 10.1136/jnnp-2015-311242 26319437

[B31] Kang S. Y. Wasaka T. Shamim E. A. Auh S. Ueki Y. Lopez G. J. (2010). Characteristics of the sequence effect in Parkinson’s disease. *J. Mov. Disord.* 25 2148–2155. 10.1002/mds.23251 PMC4782591 20669182

[B32] Kaski D. Dominguez R. O. Allum J. H. Islam A. F. Bronstein A. M. (2014). Combining physical training with transcranial direct current stimulation to improve gait in Parkinson’s disease: a pilot randomized controlled study. *Clin. Rehabil.* 28 1115–1124. 10.1177/0269215514534277 24849794

[B33] Kawashima S. Ueki Y. Kato T. Ito K. Matsukawa N. (2018). Reduced striatal dopamine release during motor skill acquisition in Parkinson’s disease. *PLoS One* 13:e0196661 10.1371/journal.pone.0196661 PMC5976194 29847548

[B34] Kojima S. Onishi H. Sugawara K. Kirimoto H. Suzuki M. Tamaki H. (2013). Modulation of the cortical silent period elicited by single- and paired-pulse transcranial magnetic stimulation. *BMC Neurosci.* 14:43 10.1186/1471-2202-14-43 PMC3621611 23547559

[B35] Lewis S. J. Foltynie T. Blackwell A. D. Robbins T. W. Owen A. M. Barker R. A. (2005). Heterogeniety of Parkinson’s disease in the early clinical stages using a data driven approach. *J. Neurol. Neurosurg. Psychiatry* 76 343–348. 10.1136/jnnp.2003.033530 15716523 PMC1739569

[B36] Molina-Luna K. Pekanovic A. Rohrich S. Hertler B. Schubring-Giese M. Rioult-Pedotti M. S. (2009). Dopamine in motor cortex is necessary for skill learning and synaptic plasticity. *PLoS One* 4:e7082 10.1371/journal.pone.0007082 PMC2738964 19759902

[B37] Muir R. B. Lemon R. N. (1983). Corticospinal neurons with a special role in precision grip. *Brain Res.* 261 312–316. 10.1016/0006-8993(83)90635-2 6831213

[B38] Muslimovic D. Post B. Speelman J. D. Schmand B. (2007). Motor procedural learning in Parkinson’s disease. *Brain* 130(Pt 11), 2887–2897. 10.1093/brain/awm211 17855374

[B39] Nitsche M. A. (2003). Level of action of cathodal DC polarization induced inhibition of the human motor cortex. *Clin. Neurophysiol.* 114 600–604. 10.1016/s1388-2457(02)00412-1 12686268

[B40] Nitsche M. A. (2011). Beyond the target area: remote effects of non-invasive brain stimulation in humans. *J. Physiol.* 589(Pt 13), 3053–3054. 10.1113/jphysiol.2011.211599 21724583 PMC3145922

[B41] Nitsche M. A. Paulus W. (2000). Excitability changes induced in the human motor cortex by weak transcranial direct current stimulation. *J. Physiol.* 527(Pt 3), 633–639. 10.1111/j.1469-7793.2000.t01-1-00633.x 10990547 PMC2270099

[B42] Nitsche M. A. Paulus W. (2011). Transcranial direct current stimulation–update 2011. *Restor. Neurol. Neurosci.* 29 463–492. 10.3233/RNN-2011-0618 22085959

[B43] Nitsche M. A. Schauenburg A. Lang N. Liebetanz D. Exner C. Paulus W. (2003). Facilitation of implicit motor learning by weak transcranial direct current stimulation of the primary motor cortex in the human. *J. Cogn. Neurosci.* 15 619–626. 10.1162/089892903321662994 12803972

[B44] Nojima I. Koganemaru S. Kawamata T. Fukuyama H. Mima T. (2015). Action observation with kinesthetic illusion can produce human motor plasticity. *Eur. J. Neurosci.* 41 1614–1623. 10.1111/ejn.12921 25892447

[B45] Nojima I. Mima T. Koganemaru S. Thabit M. N. Fukuyama H. Kawamata T. (2012). Human motor plasticity induced by mirror visual feedback. *J. Neurosci.* 32 1293–1300. 10.1523/JNEUROSCI.5364-11.2012 22279214 PMC6796271

[B46] Oldfield R. C. (1971). The assessment and analysis of handedness: the edinburgh inventory. *Neuropsychologia* 9 97–113. 10.1016/0028-3932(71)90067-4 5146491

[B47] Pelosin E. Bove M. Ruggeri P. Avanzino L. Abbruzzese G. (2013). Reduction of bradykinesia of finger movements by a single session of action observation in Parkinson disease. *Neurorehabil. Neural Repair.* 27 552–560. 10.1177/1545968312471905 23392919

[B48] Pendt L. K. Reuter I. Muller H. (2011). Motor skill learning, retention, and control deficits in Parkinson’s disease. *PLoS One* 6:e21669 10.1371/journal.pone.0021669 PMC3132742 21760898

[B49] Ramachandran V. S. Rogers-Ramachandran D. Cobb S. (1995). Touching the phantom limb. *Nature* 377 489–490. 10.1038/377489a0 7566144

[B50] Reis J. Robertson E. M. Krakauer J. W. Rothwell J. Marshall L. Gerloff C. (2008). Consensus: can transcranial direct current stimulation and transcranial magnetic stimulation enhance motor learning and memory formation? *Brain Stimul.* 1 363–369. 10.1016/j.brs.2008.08.001 PMC2621080 20633394

[B51] Reis J. Schambra H. M. Cohen L. G. Buch E. R. Fritsch B. Zarahn E. (2009). Noninvasive cortical stimulation enhances motor skill acquisition over multiple days through an effect on consolidation. *Proc. Natl. Acad. Sci. U.S.A.* 106 1590–1595. 10.1073/pnas.0805413106 19164589 PMC2635787

[B52] Ridding M. C. Rothwell J. C. (1997). Stimulus/response curves as a method of measureing motor cortical excitability in man. *Elentroencephalogr. Clin. Neurophysiol.* 105 340–344. 10.1016/s0924-980x(97)00041-6 9362997

[B53] Rizzolatti G. Fadiga L. Gallese V. Fogassi L. (1996a). Premotor cortex and the recognition of motor actions. *Brain Res. Cogn. Brain Res.* 3 131–141. 10.1016/0926-6410(95)00038-0 8713554

[B54] Rizzolatti G. Fadiga L. Matelli M. Bettinardi V. Paulesu E. Perani D. (1996b). Localization of grasp representations in humans by PET: 1. Observation versus execution. *Exp. Brain Res.* 111 246–252. 8891654 10.1007/BF00227301

[B55] Rossini P. M. Barker A. T. Berardelli A. Caramia M. D. Caruso G. Cracco R. Q. (1994). Non-invasive electrical and magnetic stimulation of the brain, spinal cord and roots: basic principles and procedures for routine clinical application. Report of an IFCN committee. *Electroencephalogr. Clin. Neurophysiol.* 91 79–92. 10.1016/0013-4694(94)90029-9 7519144

[B56] Sasaki A. T. Kochiyama T. Sugiura M. Tanabe H. C. Sadato N. (2012). Neural networks for action representation: a functional magnetic-resonance imaging and dynamic causal modeling study. *Front. Hum. Neurosci.* 6:236 10.3389/fnhum.2012.00236 PMC3418609 22912611

[B57] Thobois S. Dominey P. F. Decety J. Pollak P. P. Gregoire M. C. Le Bars P. D. (2000). Motor imagery in normal subjects and in asymmetrical Parkinson’s disease: a PET study. *Neurology* 55 996–1002. 10.1212/wnl.55.7.996 11061258

[B58] Tremblay F. Leonard G. Tremblay L. (2008). Corticomotor facilitation associated with observation and imagery of hand actions is impaired in Parkinson’s disease. *Exp. Brain Res.* 185 249–257. 10.1007/s00221-007-1150-6 17926025

[B59] von Rein E. Hoff M. Kaminski E. Sehm B. Steele C. J. Villringer A. (2015). Improving motor performance without training: the effect of combining mirror visual feedback with transcranial direct current stimulation. *J. Neurophysiol.* 113 2383–2389. 10.1152/jn.00832.2014 25632079 PMC4416593

[B60] Yamaguchi S. Kitamura M. Ushikubo T. Murata A. Akagi R. Sasho T. (2015). Effect of laterally wedged insoles on the external knee adduction moment a cross different reference frames. *PLoS One* 10:e0138554 10.1371/journal.pone.0138554 PMC4580408 26397375

[B61] Yavuzer G. Selles R. Sezer N. Sutbeyaz S. Bussmann J. B. Koseoglu F. (2008). Mirror therapy improves hand function in subacute stroke: a randomized controlled trial. *Arch. Phys. Med. Rehabil.* 89 393–398. 10.1016/j.apmr.2007.08.162 18295613

[B62] Ziemann U. Netz J. Szelenyi A. Homberg V. (1993). Spinal and supraspinal mechanisms contribute to the silent period in the contracting soleus muscle after transcranial magnetic stimulation of human motor cortex. *Neurosci. Lett.* 156 167–171. 10.1016/0304-3940(93)90464-v 8414181

